# Exploring the Relations Between Running Variability and Injury Susceptibility: A Scoping Review

**DOI:** 10.3390/sports13020055

**Published:** 2025-02-13

**Authors:** Zaheen Ahmed Iqbal, Daniel Hung-Kay Chow

**Affiliations:** Department of Health & Physical Education, The Education University of Hong Kong, Tai Po, Hong Kong SAR, China; s1136208@s.eduhk.hk

**Keywords:** running variability, movement variability, running-related injury, linear and nonlinear variability, optimal variability

## Abstract

Background: Variability in running mechanics, termed running variability, reflects the adaptability of the locomotor system to dynamic environments. Due to inconsistent findings in the literature, there is a research gap in understanding its role in injury. Objectives: This scoping review explores running variability’s influence on injury susceptibility, examining studies across various injury types, skill levels, methods, and analysis adhering to the guidelines outlined in the Preferred Reporting Items for Systematic reviews and Meta-Analyses extension for Scoping Reviews checklist (PRISMA-ScR). Eligibility criteria and sources of evidence: Twenty-one studies illustrating the complexity of running variability in relation to running-related injuries were selected from Web of Science, ScienceDirect, Google Scholar, and PubMed databases during December 2022 to June 2024. Results: There are significant differences in running variability between injured and healthy runners, with variability influenced by injury type, stage, and individual differences with varying levels of evidence. Out of 21 studies, 8 (38%) found no group differences, 11 (52%) noted higher running variability in injured participants, and 5 (24%) reported lower variability in injured than healthy subjects. The review was constrained by the diverse subjects, methods, tasks, and outcome variables across the studies. Conclusions: Currently, there is no standard normal range for running variability and abnormal variability is defined relative to control groups, though healthy controls can also show abnormal variability without injury in some study designs. Despite the absence of standardized running variability norms, wearable sensors offer insights into real-world running mechanics, presenting running variability as a potential predictor of running-related injuries. The review highlights the need for standardized protocols and further research to clarify running variability’s role in injury prediction and prevention, emphasizing the necessity of individualized approaches in training and rehabilitation. Future studies should aim to establish a causal relationship between running variability and injury susceptibility, focusing on identifying variability patterns that precede or follow an injury. This review sets the stage for developing evidence-based strategies to optimize running performance and minimize injury risks.

## 1. Introduction

Running is a skill that is developed in childhood and can be honed through practice [1]. It involves the rapid cyclical movement of the lower limbs, with multiple strides making up the intervals between consecutive strikes of the same foot [2]. The brain orchestrates the running movement based on sensory information about the environment, and runners employ various motor strategies resulting in small fluctuations between strides known as “stride-to-stride variability” (SSV) or “running variability” (RV) [3,4]. RV encompasses the natural fluctuations in running mechanics and technique that occur from stride to stride, including variances in stride length, frequency, ground contact time, foot strike pattern, and other biomechanical factors [5]. This variability is considered a reflection of the adaptability and flexibility of the locomotor system in response to dynamic internal and external environments [6,7]. Initially, researchers mainly focused on the invariant characteristics of running pattern and variation between strides was considered as noise [8]. However, RV is now recognized as a functional aspect of running [9,10].

Different researchers have proposed different types of movement variability in different contexts. RV consists of two levels: movement execution, which involves lower limb coordination variability, and movement outcome, which represents stride time variability [11,12]. Hamill, Palmer, and Van Emmerik introduced two types of variability: endpoint variability (e.g., stride time and length variability) and coordination variability (variability segmental relations) [13]. They suggested that the endpoint variability decreases with a higher level of skill [10]. Higher coordination variability increases movement pattern flexibility, allowing goal-directed task performance despite perturbations [14]. Cowin et al. proposed a theoretical framework comprising three types of movement variability: strategic, execution, and outcome [15]. Strategic variability refers to using different methods or approaches for task completion. Studies have shown that trained runners exhibit less variability compared to non-runners, with RV influenced by factors such as fatigue, terrain, running speed, and individual differences in running style and body mechanics [16]. There are conflicting results about RV and injury susceptibility. While some research suggests that higher RV may be linked to improved performance and reduced injury risk, excessive variability or significant changes in running mechanics could indicate poor form or underlying issues that may increase the risk of injury [17]. Several studies have suggested that increased RV may be associated with a decreased risk of injury, as it indicates a more adaptable and resilient gait pattern. Conversely, decreased RV may indicate a more rigid and potentially injury-prone gait pattern. With the reference to the framework of dynamic systems theory, it is proposed that decreased variability in movement may lead to progressive excessive stress on a specific joint structure, leading to injury [18,19]. Both high and low variability have been shown among subjects with patellofemoral pain syndrome (PFPS) despite using similar tools for analysis [13,20]. The relationship between RV and risk of running-related injuries (RRIs) is complex and not yet fully understood. It lacks standardization in definitions, inclusion criteria, methods, and outcome measures, leading to conflicting results and challenges in interpretation [5,21]. Since there are no proposed normative values for variability parameters, abnormal variability is currently defined as relative to the data from control groups, making it subjective and depending on individual factors like a runner’s experience, fitness level, and training goals.

A research gap lies in understanding whether altered movement variability is a cause or effect of injury. Studies show inconsistent RV findings in injured runners, with unclear mechanisms behind RRIs. A systematic review is needed to clarify these biomechanical differences and synthesize current literature findings. To the best of our knowledge, no review study has been conducted to investigate the effect of RV on injury susceptibility. This scoping review was conducted to summarize currently published comparison trials to determine whether the RV differs between injured, recovered, and healthy subjects. By examining studies investigating RV and various types of injuries including PFPS, iliotibial band syndrome (ITBS), musculoskeletal pain, instability, etc., among runners of different skill levels (recreational, professional runners, etc.), this review will consider different types of RV (kinematic, kinetic, coordination variability), methods (linear, nonlinear), and data acquisition modes (motion capture technology, wearable devices). As it is unclear why variability differs among subjects with similar type of injuries, this review explores the differential impact of the stage of injury, i.e., current injury or recovered. As a diverse range of methods has been used to measure variability, this review also provides methodological recommendations for future research. The results of this study will help to clarify the evidence on the relationship between RV and injury risk, offering insights into further research about predicting and preventing RRIs.

## 2. Materials and Methods

### 2.1. Study Design

A comprehensive scoping review was carried out to collect pertinent research on RV. The search process adhered to the guidelines outlined in the Preferred Reporting Items for Systematic reviews and Meta-Analyses extension for Scoping Reviews checklist (PRISMA-ScR) [22].

### 2.2. Search Strategy

Four databases, namely Web of Science, ScienceDirect, Google Scholar, and PubMed, were systematically searched from December 2022 to June 2024 to identify all relevant journal articles that investigate RV during running. Keywords including “RV”, “SSV”, “movement variability”, “gait”, “variability analysis”, “RRI”, “injury”, “motion capture technology”, and “inertial measurement units (IMUs)” were utilized. We also included gray literature sources like technical reports, theses and conference proceedings, etc., to obtain a more comprehensive overview of the available evidence.

### 2.3. Inclusion and Exclusion Criteria

Studies investigating RV and acute, chronic or recovered injuries that could affect the musculoskeletal system were included in this review. Running gait variability was assessed by linear or nonlinear tools or the interaction between two joints or segments denoting coordination variability. All these measures present a different aspect of RV. Full-text availability was verified, and the reference lists of identified studies were examined to locate related articles. Following the screening of titles and abstracts, duplicates and studies not aligned with the review’s scope were excluded. Studies that explored RV between injured, recovered, and uninjured controls across various running events, meeting at least level IV evidence criteria as per the Center of Evidence-Based Medicine and published in English since 2001, were included in this review. Exclusion criteria encompassed studies that did not involve original research (i.e., review articles, conference proceedings, and dissertations), studies not involving human subjects, and studies investigating neurological disorders and variability while involved in activities other than running.

### 2.4. Synthesis Methods

Synthesis methods used in this study included descriptive analysis (involving summarizing the basic characteristics of the included studies, such as study design, population, intervention, and outcomes), thematic analysis (identifying and categorizing themes or patterns that emerged from the literature), mapping (creating visual representations, such as charts, tables, or concept maps, to illustrate the distribution and characteristics of the research), narrative synthesis (providing a qualitative summary of findings, discussing the main concepts, trends, and gaps in the literature), and framework analysis (using a predefined framework to organize and interpret the data).

## 3. Results

**Figure 1:**
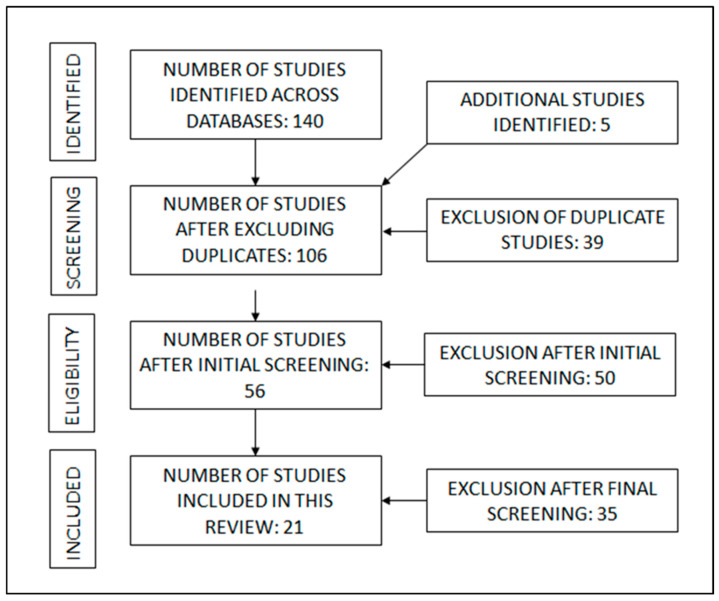
PRISMA flow diagram representing the article search process of this study.

The initial database search yielded 305 studies. After removing 39 duplicate and 50 other studies (irrelevant, without full text and conference abstracts), 216 studies remained. After screening the full texts of these studies, another 41 studies were excluded as they either provided no information on the control group, inferential statistical comparison, did not measure variability while running, or were review papers. Finally, a total of 21 studies were included in this review. Due to the heterogeneity of the data and the use of different methods and inclusion criteria among these studies, a meta-analysis was not feasible. Due to the broad and intricate nature of the topic, this scoping review aimed to outline the current main concepts without evaluating study designs [15,23]. The review followed the PRISMA guidelines (Figure 1).

The included studies were conducted in different parts of the world using different study designs including prospective longitudinal [20,24], retrospective [25,26], cross-sectional [27,28,29,30,31,32,33,34], and case-control [35,36,37,38,39,40,41,42,43]. These studies considered RV following different musculoskeletal injuries, including ligament injuries [29,35,36,38,39,40], PFPS [27,41,42,43], ITBS [28,30,37], other lower limb and RRIs [20,25,26,31,32,33,34], and musculoskeletal pain [24]. These studies quantified variability using either linear (standard deviation and coefficient of variation) [26,32,35], nonlinear (detrended fluctuation analysis and control entropy) [24] or coordination (vector coding or continuous relative phase) [20,27,28,29,30,33,34,36,37,38,39,40,41], or more than one method [25,31,42,43]. Seventeen out of twenty-one reviewed studies (81%) compared differences in RV between injured and healthy control group while seven out of twenty-one reviewed studies (33%) included subjects who had fully recovered from their injury recently or had no current symptoms. Eight out of twenty-one reviewed studies (38%) reported no significant differences between groups. Among the remaining studies, significant between-group differences in RV movement variability were reported with strong, limited, or mixed levels of evidence. Eleven out of twenty-one reviewed studies (52%) reported significantly higher RV in at least one parameter among injured subjects as compared to five out of twenty-one reviewed studies (24%) that reported lower variability as compared to healthy subjects. Details from the included studies about subject characteristics, tasks, variables, and outcomes have been presented in Table 1.

The subsequent section delves into the review’s findings, exploring the link between coordination variability, injury types and stages, and the methods for quantifying and factors influencing RV and injury susceptibility. The idea of optimal variability is presented. Additionally, the use of wearable sensors to analyze RV in real-world settings and the potential of RV as a predictor of RRIs are discussed.

## 4. Discussion

A history of injury is believed to cause abnormal biomechanics, increasing the risk of further injury [44,45]. However, it is unclear if altered movement variability causes or results from injury. Studies show inconsistent RV findings in injured runners, necessitating a systematic review to understand the mechanisms behind repetitive running injuries (RRIs). This review is one of the first to examine common movement strategies among injured subjects while running; however, results are not generalizable due to a high number of different variables included across the studies and as most of the variables used to measure variability are only used in a single study and the level of evidence is also variable.

Researchers have previously reviewed papers investigating movement variability considering factors like neurological disorders [46], knee ligament injuries [47], aging, and falls [48]. Besides running, studies measuring variability while tasks like such as jumping, landing or a cut tasks have also been reviewed [49,50]. Results from these review studies cannot be applied to running gait variability among subjects with or without RRIs. The current study is one of the few studies to review RV differences between injured, recovered, and healthy subjects. The overall findings suggest that injured subjects demonstrate an abnormal range of variability as compared to healthy subjects, with at least 13 out of 21 of the reviewed studies (62%) reporting significant differences between them. However, since no normative parameters exist for RV, both high and low variations as compared to control group have been considered ‘optimal’ deviations in motor control. It is popularly believed that rather than being beneficial, altered movement variability following an initial injury contributes to future injuries [45]. The findings of this review also challenge the variability-RRI hypothesis, which suggests that a decrease in RV would increase the risk of RRI [51], as 11 out of 21 reviewed studies (52%) reported significantly higher RV in at least one parameter among injured subjects as compared to 5 out of 21 reviewed studies (24%) that reported lower variability as compared to healthy subjects.

### 4.1. Coordination Variability

For coordination variability, altered variability in knee–ankle–foot and trunk–pelvis couplings, that are used in more than one study, are most commonly reported among injured runners [27,30,39,41,43]. Variability of shank–rearfoot, thigh–shank couplings, and stride time while running revealed no between-group differences [25,28,29,30,31,36,40]. Results suggest that RV strategies between injured and healthy subjects differ in particular joint couplings, the most common being knee–ankle–foot joint coupling, with both high and low relative variability demonstrated among injured runners [27,30,39,43]. Variability in shank–rearfoot coupling in subjects with chronic ankle instability and ITBS was not statistically different from that of healthy subjects, suggesting that coordination variability in this coupling is similar among adults irrespective of injury [28,29,30,36,40]. Other studies that also examined other couplings in addition to shank–rearfoot coupling reported altered variability for knee–ankle and thigh–shank couplings suggesting that alterations in variability due to injury tend to occur in more proximal parts while running [30,39,40]. Runners with PFPS exhibited lesser trunk–pelvis variability as compared to healthy counterparts [41]. Lower trunk–pelvis variability has also been reported among subjects with pain while walking, suggesting that it is also present at slower speeds [41,52].

The relationship between RRI and coordination variability has been widely explored [13]. Different methods, such as modified vector coding and continuous relative phase, have been used to assess coordination variability [53]. Studies have reported that runners with certain injuries exhibit less coordination variability [39,54]. Lower coordination variability indicates a loss of complexity in the locomotor system and may increase the risk of RRIs [55,56]. However, findings regarding the relationship between coordination variability and RRI have been inconsistent [28,30]. Factors such as gender, age, running experience, fatigue, and biomechanical factors can affect coordination variability during running and cause inconsistent findings [28,57,58,59]. Lower limb coordination is crucial in understanding running mechanics and the risk of RRIs. Fatigue progression and running experience can affect lower limb coordination during long runs [57], but the findings regarding their effects on running mechanics have also been inconsistent [60,61,62]. The coordination patterns of novice and experienced runners differ, suggesting that running experience affects coordination variability. Anaerobic threshold speed is the most effective physiological indicator of running performance, and runners are recommended to train at this speed to improve their performance. However, studies on lower limb coordination have only conducted running tests at comfortable or slow fixed speeds [57,58,63]. Another study by Mo and Chow investigated the coordination variability and lower limb coordination patterns of novice and experienced runners during prolonged treadmill running at their anaerobic threshold speed [11]. Their results indicated that running experience and fatigue can affect lower limb coordination patterns and that the runner groups exhibited different coordination patterns. Coordination variability has been proposed as a tool for identifying skill-dependent factors affecting sports performance.

### 4.2. Type and Stage of Injury

Higher variability was reported among subjects with ITBS and PFPS while lower variability was reported in subjects with chronic ankle instability [27,30,39,43]. This further suggests that type and location of injury has a differential effect on RV. There is an indication that higher or lower variability could be observed among injury-prone subjects [15], which needs to be further investigated. The current study also examined whether the stage of injury has any differential effect on RV. Seventeen out of twenty-one studies (81%) reviewed in this study compared differences in RV between injured and healthy control groups while seven out of twenty-one reviewed studies (33%) included subjects who fully recovered from their injury recently and had no current symptoms. As other studies have shown that performance of functional tasks is altered in patients with current pain, this suggests that altered RV may be a compensation for symptoms of injury [64,65]. Symptoms from injury signal the neuromuscular system to adopt alternative movement strategies to decrease pain and protect body structures from further injury [66,67]. Such alternate movement strategies bring short-term benefits to continue the task; however, if continued for a longer period the higher or lower relative variability increases the risk of further injuries [13,20]. As most of the current injuries considered in this review were chronic, i.e., more than 3 months old, it is possible that maladaptive alternate movement strategies had been adopted over time. Two of the reviewed studies identified a potential relationship between altered RV and prospective RRI among runners, which further indicates that altered variability precedes injury [20,24].

Only three out of seven studies (43%) that considered subject who recovered their injuries (mainly PFPS and ITBS) were found to significantly affect RV [25,30,40]. Subjects recovered from other RRIs did not report significant effects [25,28,30,31,32]. It is also interesting to note the differences in RV between the injured and recovered subjects. This further points towards the persistence of altered movement strategies even after the resolution of injury in some injury cases [5,68,69]. Again, heterogeneity among RRIs and its duration can mask the differences between injured and healthy subjects, and further prospective research is needed to determine the effect of altered RV before, during, and after sustaining injuries.

### 4.3. Linear and Nonlinear Methods

Only two out of twenty-one studies (9%) that were reviewed used nonlinear methods to analyze RV. Traditionally, stride interval variability has been quantified using linear methods, but these methods may not fully capture its temporal nature [5]. In fact, one of the review studies about lower extremity movement variability between injured and heathy subjects specifically excluded studies that used nonlinear measures to examine variability, as there are fewer studies on the structure of variability that prevent drawing any conclusion [49]. Nonlinear methods, such as detrended fluctuation analysis, have revealed fractal long-range correlations in stride interval variability [4,12,70]. Various factors, such as health status, fatigue, footwear, running speed, and foot strike pattern, have been explored in relation to stride interval variability [10,25,31,71,72,73]. A study by Mo and Chow reported differences in long-range correlations and stride interval variability magnitude among novice and experienced runners while running for a long period on a treadmill at their personal anaerobic threshold speed [74]. These findings suggest that running experience and fatigue can affect stride interval variability. However, more studies are needed to understand the relation between injury, duration, and surface of running in the natural environment outside the laboratory.

### 4.4. Factors Affecting Running Variability

Studies have shown that factors such as cadence and footwear can affect the variability of gait in runners with injuries like PFPS [75]. As stride interval dynamics can be affected by different factors, such as running experience, speed, shoes, surface, and fatigue, variation in participant characteristics and study designs may contribute to these inconsistencies [31,71,72,73,76,77]. Other factors, such as task difficulty and variability of interest, could also contribute to the inconsistent differences in the magnitude of the stride interval variability between injured and healthy individuals [32,78].

Research indicates that lower limb joint angles and their variability during running are affected by factors like running duration and the surface [77]. Notably, joint angle variability is greater while running over ground rather than on a treadmill. Initially, when fatigue is low, joint angle variability is higher, but it decreases as running continues. During this time, joint angles, especially in the hip and ankle, increase, while knee motion range decreases, particularly during over-ground running. These changes might help achieve longer strides in distance running. While treadmill running offers a good simulation, it might not fully capture outdoor dynamics. Thus, these variations should be considered in gait analysis and when designing sports training and rehabilitation programs. Understanding the variability in lower limb joint angles during running can be crucial in linking running mechanics to injury susceptibility. Variability, especially in over-ground running, may indicate how the body adapts to different surfaces and conditions. High variability in joint angles might suggest an increased ability to adapt, potentially reducing injury risk by preventing repetitive stress on specific joints. Conversely, decreased variability, especially as fatigue sets in, might lead to a more rigid running form, increasing the likelihood of overuse injuries. By analyzing these patterns, trainers and therapists can develop personalized training and rehabilitation programs to enhance adaptability and minimize injury risks.

### 4.5. Optimal Variability

**Figure 2:**
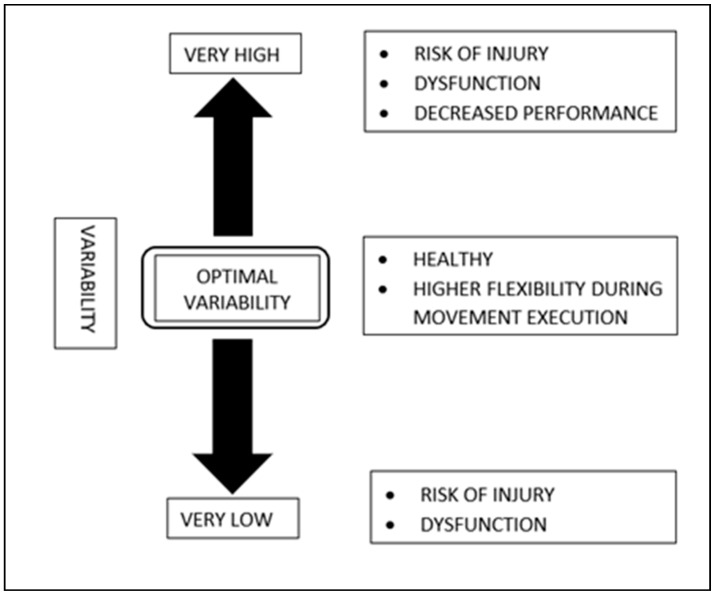
Diagrammatical representation of highly complex optimal variability. Very high and very low variability reduce the functional capacity of the system, leading to injury, dysfunction, and poor performance. Hypothetical optimal variability in movement systems indicates stability, flexibility, and a healthy status.

The role of variability in RRIs is not clearly understood as both high and low movement variability can be detrimental to running performance [6,13,79]. This raises the question of whether an optimal variability that maximizes training results while minimizing injury risk exists [13,68]. The concept of optimal movement variability suggests a balance between predictability and complexity exists [80,81]. Coordinated skills, like walking and running, should strike a balance between these two extreme situations to fit within what is implied as optimal movement variability [82]. Understanding the optimal level or range of movement variability may help assess rehabilitation outcomes and identify injury risks. Thus, the notable concern is whether and how this optimal variability level or range can be investigated. In 2012, Hamill et al. proposed the loss-of-complexity hypothesis for injury or pathology [13]. This hypothesis suggests that reducing movement complexity within functional synergies would increase the risk of injury or disease. In this case, an individual would be clinically affected because the overall functional capacity of their locomotor system would decrease to below the critical threshold. Although strong associations between altered motor system variability and injury or dysfunction have been suggested, the causal relationship between them has yet to be verified. Whether a change in motor system variability occurs as an affliction is unclear. This relationship to be confirmed, even if not causal, would indicate that motor system variability affects disease or injury risk [83]. This interpretation is further complicated by the numerous factors that can affect SSV and the conflicting findings regarding the effects of RV on RRI development. However, the factors affecting movement variability and its effects on RRI development are still unclear [84]. Research is required to understand further the effects of various factors, such as running experience and performance, level of training, distance and surface of running, participation in running events, and progressive fatigue, on the RV among healthy runners. The concept of optimal variability is presented in Figure 2.

### 4.6. Wearable Sensors

The relationship between RRI and task outcome variability remains unclear. Stride interval is associated with running performance and efficiency, making it a crucial parameter for exploring running biomechanics [85]. However, gait variability has been extensively studied in relation to locomotor control during short-duration running inside the laboratory using motion capture systems. Due to the limitations of motion capture systems, 19 out of 21 studies (90%) were conducted inside laboratory, which cannot completely replicate the natural demands on the body while outdoor running. Only 2 out of 21 studies (9%) that were reviewed used accelerometers for data acquisition. With the wide availability of wearable sensors, investigating RV outside the laboratory in natural environment suggests the future research directions. Wearable devices that can track simple metrics can provide similar feedback on running mechanics in a realistic on-field environment [86,87]. Distance runners show more variability during the swing phase and in knee joints compared to other lower body joints. High-mileage runners exhibit consistent joint kinematics and angle variability, indicating strong correlations among joints and phases in the sagittal plane [88]. Therefore, distance runners, coaches, researchers, and clinicians should consider joint angle variability along with movement path analysis.

### 4.7. Running Variability as a Predictor of RRIs

RV can potentially predict RRIs. Although models of relationship between the training and performance have been constructed for individual sports athletes [89,90], there is no proposed model for relationship between RV and susceptibility of injury. Monitoring changes in running metrics such as stride length, cadence, ground contact time, and other biomechanical parameters can provide valuable insights into an athlete’s risk of injury [91]. While RV can provide valuable insights into an athlete’s injury risk, it is important to note that predicting injuries is complex and multifactorial. Other factors such as training history, biomechanics, muscle strength, flexibility, and overall health should also be considered when assessing injury risk. Using wearable sensor technology in conjunction with data analytics can help to identify as well as mitigate the risk of injury among runners [92]. Some research studies have explored the use of RV data in developing injury prediction models [92]; however, a multidimensional approach with involvement of professionals from sports medicine and physical therapy are required for a comprehensive assessment and for more accurate data to train and test such models. By collecting and analyzing data on running metrics and injury history, researchers can identify patterns and trends that may help predict the likelihood of specific injuries in athletes. These models can be used to inform injury prevention strategies and guide training modifications to reduce the risk of injury [93]. Overall, RV can serve as a valuable tool in predicting and preventing RRIs by providing insights into biomechanics, movement patterns, training load management, and injury prediction models. By monitoring changes in running metrics and addressing potential risk factors early on, athletes and coaches can take proactive measures to reduce the risk of injury and optimize performance.

### 4.8. Limitations

Studies included in this review adopted a standard approach to analyze differences in RV at the group level. Such analyses, i.e., comparing the mean difference in outcomes of variability, could potentially overlook the nature of individual variability responses. Analysis of intra-subject variability differences before and after sustaining the injury may be more relevant in identifying factors that cause the changes in RV and its potential clinical implications. Due to the heterogeneity of subjects, running tasks, methodologies, and outcome variables among the studies included in this review, it was limited to a summative synthesis of the literature. Since there are no proposed normative values for variability parameters, abnormal variability is currently defined as relative to the data from control groups; however, in retrospective or cross-sectional study designs, healthy subjects in the control group can also present abnormal variability without developing injury.

### 4.9. Practical Applications and Future Research Directions

The findings from the current review highlight important practical applications and future research directions in understanding RV and its relationship with RRIs. Practically, this research underscores the need for personalized rehabilitation and training programs that account for individual variability in biomechanics, aiming to optimize movement patterns and reduce injury risk. Wearable sensors could play a crucial role in monitoring RV in real-world settings, offering insights into runners’ biomechanics and aiding in injury prevention strategies. Future research should aim to establish standard values for RV to clarify what “optimal” variability involves. Longitudinal studies are crucial in examining how RV evolves over time concerning injury onset and recovery, and for determining whether changes in RV are a cause or consequence of injuries. Additionally, understanding the effects of factors like running experience, fatigue, and environmental conditions on RV can offer deeper insights into injury mechanisms. Interdisciplinary collaboration, combining biomechanics, sports medicine, and data analytics, will be vital for progress in this area. More prospective studies are needed to enhance evidence on how various injuries impact RV. This review’s findings should guide future investigations into how injuries affect RV, addressing the current variability in outcome measures.

## 5. Conclusions

This review indicates that RV among injured or recovered individuals often differs from that of healthy runners, with a general trend toward greater RV in injured subjects. However, this trend is not uniform across all injury types and stages. The absence of a universally accepted normal range for RV stems from the complexity and individuality of running mechanics, influenced by factors like biomechanics, experience, and environmental conditions. Developing standardized parameters and protocols for RV measurement, possibly using wearable sensors, could help establish norms and identify optimal variability levels that minimize injury risk. However, these norms must accommodate individual differences in anatomy, fitness, and training history. Thus, while standardization can offer general guidelines, personalized approaches remain essential. Future studies are needed to explore the cause-and-effect relationship between RV and injury risk, determining whether RV changes precede or follow an injury to identify high-risk runners.

## Figures and Tables

**Table 1:** Characteristics and summary results for included studies comparing running variability and injury.

Study	Study Design	Mode of Running/Speed (Km/h)	Data Acquisition: Frequency (Hz)	Population (Age, Years)	Type of Injury	Gender Sample Size	Variability Analyzed: Technique/Metric	Result
**Linear Variability**								
[35]	Case-Control	Running on Treadmill/Fixed (10)	3D Motion Analysis: 200	Recreational Athletes (24 ± 3)	Chronic Ankle Instability-Inversion Sprain	Mixed Injured: 12, Control: 12	Joint Angles: Standard Deviation	Higher Variability in Injured Group
[32]	Cross-Sectional	Running on Treadmill/Self-Selected (Recovered: 10.4, Control: 11.2, 75% Maximum)	3D Motion Analysis: 240	Recreational Runners (28 ± 7)	Running-related Injuries	Mixed Recovered: 23, Control: 21	Joint Angles: Standard Deviation	No Significant Difference
[26]	Retro-spective Cross-Sectional	Running on Treadmill/Self-Selected (Control: 6.6, Recovered: 7.2, Injured 7.3)	Zebris FDM-T Treadmill: 120	Runners (30 ± 5)	Running-Related Injuries	Mixed Injured: 16 Recovered: 13, Control: 14	Navicular Drop Height, Ground Reaction Forces Midfoot Pressure, and Foot Rotation Angle: Standard Deviation	Higher Variability in Injured Group
**Nonlinear Variability**								
[24]	Prospective Longitudinal	Running Over Different Over-Ground Surfaces/Slightly Faster Than Preferred (13.3)	Accelero-meter: 100	Collegiate Cross-Country Runners (19.79 ± 1.35)	Musculo-skeletal Pain	Mixed Injured: 7, Control: 15	Center of Mass Acceleration Complexity: Control Entropy	No Significant Difference
**Coordination Variability**								
[33]	Cross-Sectional	Running on 25 m Laboratory Runway (13.14)	3D Motion Analysis: 120	Runners (29.9 ± 12.2)	Various Lower Extremity Injuries	Mixed Injured: 11, Control: 11	Coordi-nation: Vector Coding	No Significant Difference
[30]	Cross-Sectional	Running on Treadmill/Self-Selected (Recovered: 10.2, Control: 12.4)	3D Motion Analysis: 120	Recreational Runners (27.5 ± 9)	Iliotibial Band Syndrome	NR Recovered: 8 Control: 8	Coordi-nation: Continuous Relative Phase	Lower and Higher Variability in Injured Group
[36]	Case-Control	Walking and Jogging on Treadmill/Fixed (9.7)	3D Motion Analysis: 120	University Students (24.6 ± 4.2)	Chronic Ankle Instability-Inversion Sprain	Mixed Injured: 7, Control: 7	Coordi-nation: Continuous Relative Phase	No Significant Difference
[34]	Cross-Sectional	Running, Treadmill/Fixed (10.8)	3D Motion Analysis: 240	Runners (NR)	Overuse Knee Injury	Females Injured: 9, Control: 9	Coordi-nation: Modified Vector Coding	Higher Variability in Injured Group
[37]	Case-Control	Running on EVA Foam Laboratory Runway (11.8)	3D Motion Analysis: 250	Recreational Runners (37 ± 9)	Iliotibial Band Syndrome	Females Injured: 18 Control: 18	Coordination: Continuous Relative Phase	No Significant Difference
[27]	Cross-Sectional	Running on Treadmill/Self-Selected (Injury: 10.4, Control: 9.1)	3D Motion Analysis: 300	Recreational Runners (25 ± 6.1)	Patello-femoral Pain	Females Injured: 19, Control: 11	Coordination: Vector Coding	Higher Variability in Injured Group
[29]	Cross-Sectional	Walking and Jogging on Treadmill/Fixed (4.83 and 9.66)	3D Motion Analysis: 250	Recreational Athletes (26.9 ± 6.8)	Chronic Ankle Instability	Mixed Injured: 15, Control: 13	Coordination: Vector Coding	No Significant Difference (Jogging)
[38]	Case-Control	Walking and Running on Treadmill/Self-Selected (4.83 and 9.66)	3D Motion Analysis: NR	Recreational Athletes (22.5 ± 5)	Anterior Cruciate Ligament Recon-struction	Mixed Injured: 22 Control: 15	Coordination: Vector Coding	Higher Variability in Injured Group
[28]	Cross-Sectional	Running on Treadmill/Self-Selected (Injury: 10.8, Control: 11.9)	3D Motion Analysis: 8	Recreational Runners (30.2 ± 7)	Iliotibial Band Syndrome	Females Injured/ Recovered: 13, Control: 12	Coordi-nation: Vector Coding	No Significant Difference
[39]	Case-Control	Walking and Jogging on Treadmill/Fixed (4.83 and 9.7)	3D Motion Analysis: 250	Recreational Runners (23.8 ± 5.1)	Chronic Ankle Instability -Inversion Sprain	Mixed Injured: 13 Control: 14	Coordi-nation: Vector Coding	Lower Variability in Injured Group
[40]	Case-Control	Running on Treadmill/Fixed (9.6)	3D Motion Analysis: 120	General Population (22.3 ± 2.5)	Chronic Ankle Instability—Ankle Inversion Sprain	Mixed Injured: 17 Recovered: 17 Control: 17	Coordination: Vector Coding	Lower and Higher Variability in Injured/ Recovered Groups
[41]	Case-Control	Walking and Running on Treadmill/Self-Selected (Injury: 17.6, Control: 8.0)	3D Motion Analysis: 200	General Population (25.9 ± 3.9)	Patello-femoral Pain	Female Injured: 17 Control: 17	Coordi-nation: Continuous Relative Phase	Lower Variability in Injured Group
[20]	Prospective Longitudinal	Running Over-Ground/Slightly Faster Than Preferred (13.3)	3D Motion Analysis: 240	Recreational Runners (32.3 ± 11.6)	Running-related Overuse Injuries	Mixed Injured: 21 Control: 18	Coordi-nation: Modified Vector Coding	Higher Variability in Injured Group
**Linear and Coordination Variability**								
[42]	Case-Control	Running on Treadmill/Self-Selected (Injury: 9.3, Control: 9.8)	3D Motion Analysis: 240	General Population (24 ± 6)	Patello-femoral Pain	Females Injured: 8 Control: 8	Spatio-temporal Parameters: Coefficient of Variation Coordi-nation: Vector Coding	Higher Variability in Injured Group Lower Variability in Injured Group
[43]	Case-Control	Running on Treadmill/Self-Selected (Injury: 7.1, Control: 7.7)	3D Motion Analysis: NR	Recreational Runners (21 ± 0.6)	Patello-femoral Pain	Female Injured: 6 Control: 6	Knee Joint Angles: Standard Deviation Coordi-nation: Continuous Relative Phase	Higher Variability in Injured Group Higher Variability in Injured Group
**Linear and Nonlinear Variability**								
[31]	Cross-Sectional	Running Over-Ground/Self-Selected (Recovered: 12.5, Control: 12.6)	Uni-axial Accelero-meter and BioRecorder: 1000	Recreational Runners (29.3 ± 10.3)	Running-related Injuries	NR Recovered: 9 Control: 9	Spatio-temporal Parameters: Coefficient of Variation and Detrended Fluctuation Analysis	No Significant Difference
[25]	Retro-spective Case-Control	Running on Treadmill/Self-Selected (Recovered: 10.8, Control: 10.5, 80–120%)	Runalyser—Pressure Sensitive Insole System: NR	Recreational Runners (40 ± 10)	Running-related Injuries	Mixed Recovered: 44 Control: 46	Spatio-temporal Parameters: Coefficient of Variation Strike Index: Detrended Fluctuation Analysis	No Significant Difference Higher Variability in Injured Group

(NR: not reported).
